# How Do Young Community and Citizen Science Volunteers Support Scientific Research on Biodiversity? The Case of iNaturalist

**DOI:** 10.3390/d13070318

**Published:** 2021-07-13

**Authors:** Maria Aristeidou, Christothea Herodotou, Heidi L. Ballard, Lila Higgins, Rebecca F. Johnson, Annie E. Miller, Alison N. Young, Lucy D. Robinson

**Affiliations:** 1 Institute of Educational Technology, The Open University, Milton Keynes, Buckinghamshire MK7 6AA, UK; 2 Center for Community and Citizen Science, UC Davis School of Education, University of California Davis, Davis, CA 95616, USA; 3 Natural History Museum of Los Angeles County, Los Angeles, CA 90007, USA; 4 California Academy of Sciences, San Francisco, CA 94118, USA; 5 Natural History Museum, Cromwell Road, London SW7 5BD, UK

**Keywords:** data quality, community science, citizen science, iNaturalist, online participation, young volunteers, biodiversity

## Abstract

Online community and citizen science (CCS) projects have broadened access to scientific research and enabled different forms of participation in biodiversity research; however, little is known about whether and how such opportunities are taken up by young people (aged 5-19). Furthermore, when they do participate, there is little research on whether their online activity makes a tangible contribution to scientific research. We addressed these knowledge gaps using quantitative analytical approaches and visualisations to investigate 249 youths' contributions to CCS on the iNaturalist platform, and the potential for the scientific use of their contributions. We found that nearly all the young volunteers' observations were 'verifiable' (included a photo, location, and date/time) and therefore potentially useful to biodiversity research. Furthermore, more than half were designated as 'Research Grade', with a community agreed-upon identification, making them more valuable and accessible to biodiversity science researchers. Our findings show that young volunteers with lasting participation on the platform and those aged 16-19 years are more likely to have a higher proportion of Research Grade observations than younger, or more ephemeral participants. This study enhances our understanding of young volunteers' contributions to biodiversity research, as well as the important role professional scientists and data users can play in helping verify youths' contributions to make them more accessible for biodiversity research.

## Introduction

### 1.1 Public Participation in Scientific Research

With the burgeoning interest in whether and how members of the public can effectively contribute to biodiversity research has come increasing interest in, but a lack of research evidence on, the role that young people can play in this effort. In our investigation of youth participation in biodiversity research, we use the term community and citizen science (CCS) to include the range of ways and roles that members of the public can participate in scientific research, which can include different levels of collaboration between scientists and the public, including citizen science and community science [1]. The Citizen Science Association defines citizen science as the 'involvement of the public in scientific research—whether community-driven research or global investigations' (https://www.citizenscience.org, accessed on 1 February 2021). The involvement of the public in the scientific endeavour has given rise to the 'democratisation' of science by welcoming participants of different age, gender, class and education levels [2] and inviting the contribution of diverse perspectives for a more robust science [3]. This increase in diversified participants in CCS was aided by the rapid growth of projects that use new technologies [4], such as smartphone apps.

Studies exploring the participation of adults in online CCS projects focus on and characterise volunteers' contribution behaviour. For instance, volunteers are categorised as 'high' and 'low' contributors, according to their number of contributions [5]; 'persistent' or 'hardworking', according to their participation patterns [6]; or 'careful annotation' and ’star specialisers' according to the main tasks they are involved in [7]. Further research characterising participation [6,8] reports asymmetrical participation, with a few volunteers making the majority of contributions.

### 1.2 Young People’s Participation in Community and Citizen Science

Increasing interest in involving young people in community and citizen science (CCS) largely stems from the potential and emerging evidence for fostering science learning through participation in science [1,8,9]. However, a crucial piece of the puzzle if we are to determine the ways in which young people contribute to biodiversity science is the nature and patterns of their participation, regardless of the learning that occurs. For example, in field-based settings, Lorke et al. [10] found that young people could potentially contribute to the data collection of Bioblitzes at different levels (exploring, observing, identifying organisms, documenting and recording). In terms of the participation of young people in online CCS, studies have been conducted to explore the participation profiles of young volunteers, their contributions and learning outcomes. For instance, Herodotou et al. [11] examined the participation profiles of young volunteers on Zooniverse. They found that slightly over a third were 'lasting' participants (users who remain loyal to Zooniverse), another third were 'visitors' (users who only contributed for one or two days), and only a small percentage were systematic with high activity over an extended time. Furthermore, Herodotou et al. [11] identified an asymmetrical participation pattern, similar to that of adults (e.g., [6]), where a few volunteers make the majority of contributions and the majority of them contribute only once or twice. Aristeidou et al. [12] studied the contributions of 183 young volunteers on iNaturalist and found that, compared to the observation behaviour of all iNaturalist users, they observed fewer plants and birds, and more molluscs, arachnids and insects. Moreover, an increased number of contributions of young iNaturalist volunteers were found to be associated with systematic participation and a large proportion of active days. Given insights from this study, our first hypothesis (H1) is that young volunteers are as likely to contribute verifiable and Research Grade observations as other volunteers on iNaturalist.

### 1.3 Biodiversity-Focused Community and Citizen Science

Biodiversity loss or the loss of biological diversity (for example, the reduction or extinction of species) is a wicked conservation issue that involves many different stake-holders with frequently conflicting interests and perceptions of the problem. Sharman and Mlambo [13] explained that a major challenge when tackling biodiversity loss is people’s ignorance about it or their perception that it is not an issue affecting them directly but rather a distant concern. They stress the importance of personal responsibility and individual ownership for tackling biodiversity loss. McKinley et al. [14] analyse how CCS can be a powerful tool for tackling many conservation challenges by building scientific knowledge and encouraging public action. Powney and Isaac [15] discuss the application of volunteer-collected biological records for conservation science, in particular how the voluntary nature of their collection results in longer-term datasets across greater spatial extents than would be feasible through more traditional professional surveys. Furthermore, a number of studies (e.g., [1,16]) highlighted the importance of CCS in supporting young people to become more agentic and develop capacity to take action.

Biodiversity-focused CCS engages people in collecting biodiversity data, and identifying and monitoring biological diversity [17]. In a review of CCS contributions to biodiversity, Chandler et al. [18] found the majority of biodiversity CCS projects have a narrow taxonomic focus and only collect data on insects (24%), birds (19%), plants (17%), lepidopteran insects (12%), or mammals (9%), while only 20% collect data on multiple taxonomic groups. The majority of new species occurrence data (2010-2021) shared with GBIF comes from two CCS platforms, eBird (63% of all GBIF data and 78% of all GBIF bird data) and iNaturalist (3% of all GBIF data and 11% of all GBIF non-bird data) (www.gbif.org, accessed on 23 April 2021). On both of these platforms, only species observations verified by community members as correctly identified are shared with GBIF. Within iNaturalist, community verification results in an observation being deemed 'Research Grade', and it is these observations only that are shared with GBIF (see Section 2.1.2 data quality for details). Data can be directly downloaded from GBIF and used by researchers, without those researchers ever engaging with the CCS projects that gathered them. During the period 2020−2021, 728 papers were published using eBird (106) and iNaturalist (672) data accessed through GBIF (www.gbif.org, accessed on 23 April 2021). In addition to the contributions to biodiversity research, CCS projects also have the potential to develop positive behaviour changes that can directly benefit biodiversity locally (e.g., decreased pesticide use) and to engage the public in biodiversity learning [8].

### 1.4 Data Quality in Community and Citizen Science

For data gathered through CCS to be used in scientific research, there must be a level of confidence in the accuracy and quality of the data. Data gathered by beginners and young people are often called into question for their accuracy and overall quality [19]. As a consequence of the expansion of scientific research to include non-specialists, data quality, which is a key issue in CCS, is increasingly discussed. As a response to the data quality issue in CCS, Kosmala et al. [20] argue that successful CCS projects are expected to develop methods for ensuring data accuracy and account for bias. Thus, each project dataset should be assessed individually. These methods include the iterative development of task and tool design, volunteer training and testing, standardised and calibrated equipment, expert validation, replication and calibration across volunteers, the skill-based statistical weighting of volunteer classifications and accounting for random error and systematic bias. As reported by Wiggins and Crowston [21], the most common mechanism for data validation is 'expert review'. More recent data validation and verification techniques are the 'automatic quality assessment' via software-based systems that automatically carry out a quality assessment of the collected data and hybrid techniques combining expert and machine-induced models (e.g., [22]). Particularly of note, for this study, expert validation on iNaturalist occurs when knowledgeable taxon specialists (professional or otherwise) verify observations through data quality assessment and identification which helps observations become 'Research Grade' (see Section 2.1.2).

The systematic literature review of 71 studies assessing aspects of data quality and the effect of volunteer characteristics in CCS projects found that most studies show that volunteers collect, or have the potential to collect, useful, high-quality data [23]. Some speculate that this may vary across participants with different characteristics; in a systematic review of volunteer characteristics with respect to data quality, Lewandowski and Specht [23] report that volunteer characteristics such as prior knowledge, repetition of method, training, and male volunteers were related to good data quality levels, while age and group size did not relate. While most of the related volunteer characteristics make intuitive sense, the particular study’s researchers explain that findings regarding gender as a consequence of female participants being more hesitant to perform certain tasks that would improve the data quality [24]. Other research on data quality and participation in CCS associates an increased self-identified comfort level with successful species identification [25], high levels of participation with increased numbers of correct organism identifications [26], and increased participation with reduced errors in uploading project data [27]. These studies led to our additional research questions: Do the levels of participation of young volunteers relate to the number of observations submitted to iNaturalist that become Research Grade? Does the gender or age of young volunteers relate to data quality? Concerning young people’s contributions to CCS, there is considerable ambiguity with regard to obtaining accurate data. Schuttler et al. [28] engaged young people, as young as nine years old, in using camera traps to collect mammal monitoring data and found that 94% of the camera traps were set in accordance with scientific protocols and that the generated data had community-wide impacts. However, Miczajka et al. [29], who also engaged young people (elementary school children) in measuring vegetation cover and height, found that there was some significant difference in the estimation and measurements between children and scientists—which is to be expected given the comparison between professionals and children. They concluded that it is possible to involve them in CCS activities, if the tasks require skills already acquired by children. Therefore, it is imperative for CCS project managers to design youth projects with their target audience’s skill level in mind, provide adequate training and build-in data quality assurance and control procedures.

### 1.5 Aims of This Research

The main aims of this research were to explore the extent to which young volunteers' contributions on iNaturalist (www.inaturalist.org, accessed on 1 February 2021) are of potential scientific use to biodiversity research (defined as observations that are verifiable and then further become 'Research Grade' on the platform), and how young volunteer (aged 5-19) characteristics relate to those Research Grade contributions. In particular, we followed the following research objectives (RO) and associated null hypotheses (H0):

- RO1: To explore the verifiability and quality grade of young volunteers' contributed observations, overall and per iNaturalist’s taxon category;
- RO2: To explore the relationships (if any) between participation behaviour (proportion of active days and duration) and proportion of contributed Research Grade quality observations.

**Hypothesis 1 (H1).** *iNaturalist young volunteers' participation behaviour will not be related to the proportion of contributed Research Grade observations.*

- RO 3: To explore the relationships (if any) between background characteristics, including age and gender, with the proportion of contributed Research Grade observations.

**Hypothesis 2 (H2).** *There will be no gender differences in the proportion of Research Grade observations of iNaturalist young volunteers.*

**Hypothesis 3 (H3).** *There will be no age differences in the proportion of Research Grade observations of iNaturalist young volunteers.*

This study is part of the LEARN CitSci project, an international research collaboration between three Natural History Museums (NHMs) and three research institutions in the UK and US, aiming to study young people’s participation and learning in online and field-based CCS settings and to improve the design of CCS programmes and projects offered by NHMs. All three NHMs use and/or promote iNaturalist as their primary online CCS platform for collecting biodiversity data. The findings of this study add to the limited knowledge of how and which young people contribute to biodiversity sciences through CCS.

## 2 Materials and Methods

### 2.1 iNaturalist: Contributing to Biodiversity Research

iNaturalist is one of the largest online CCS initiatives for naturalists. It holds approximately 58 million verifiable (has evidence, correct date and location, and is not marked as a captive or cultivated organism) observations of 320,000 different species made by 1,460,000 users at the time of writing (April 2021). Observations are marked as 'Research Grade' when there is at least ^2^/3 community consensus on a precise identification (usually species-level), and it is these observations that are automatically deposited to GBIF. These data are available to be used in biodiversity and other research both from GBIF and directly downloadable from iNaturalist.

The primary activities users take part in on iNaturalist are making and uploading observations of organisms and identifying the organisms in other members' observations. 'Observations', which are usually photos of a single organism submitted by a user, are annotated with metadata including date, time, location, whether the organism is captive/cultivated, taxonomic identification and other user-defined data fields. These can be particularly useful to scientists; for example, they can help estimate species distribution, develop species checklists, describe new species, and track invasive species (example below). User profile pages are open to the public, and aggregated data are available for downloading by scientists, researchers, and other members of the public.

The species images (photos), combined with a wealth of metadata and the recorded data quality validation, have attracted biodiversity scientists to use iNaturalist in their research. For example, Michonneau and Paulay [30] used iNaturalist to study the diversity of echinoderms; Rossi [31] examined red mangrove observations to detect foliar disease symptoms such as lesions; Heberling and Isaac [32] used iNaturalist to facilitate plant specimen collection and curate field images alongside physical specimens; and Putman et al. [33] used iNaturalist to quantify lizard ecological interactions in urban areas.

Moreover, the iNaturalist community aids the discovery of non-native and invasive species. Vendetti et al. [34] documented five new introduced terrestrial gastropods in Southern California, including the first U.S. record of the common chrysalis snail; Jones et al. [35] documented the discovery of painted-hand mudbugs, a new species in Canada; Liebgold et al. [36] reported the detection of mourning geckos, a non-native invasive species in the Caribbean; and Moulin [37] recorded the presence of giant Asian mantis for the first time in France.

#### 2.1.1 Taxonomic Coverage of iNaturalist

iNaturalist’s taxonomy includes all living things at all taxonomic ranks but features the following 'iconic' taxon categories, which we will use for the purposes of this paper: birds; protozoans; mammals; molluscs; insects; plants; reptiles; fungi and lichens; arachnids; ray-finned fishes; and amphibians. Examining the publicly available data on the website (21 October 2020), we found that the most observed organisms on iNaturalist are plants (42%), followed by insects (26%) and birds (14%). The least observed are protozoans, with 71,000 observations (0.1%). Unknown organisms (those currently unidentified) amount to 0.9% in iNaturalist [38].

#### 2.1.2 Research Grade Observations

Observations uploaded to iNaturalist are considered 'verifiable' (i.e., eligible to be verified and become 'Research Grade') if the observation includes evidence of an organism (a photo or a sound recording), an accurate date on which the organism was observed, and an accurate location where the organism was observed; furthermore, verifiable observations must not be marked as captive/cultivated. Verifiable observations uploaded to iNaturalist are automatically given a 'Needs ID' quality grade, a term reflecting the need for more iNaturalist community members to add identifications to bring the observation to 'Research Grade'. Observations that are uploaded without evidence of the organism, a date or location, or those that are marked as captive/cultivated are automatically given a 'Casual' quality grade and cannot become 'Research Grade' unless the observer adds the missing information, or the captive/cultivated status is overturned.

'Research Grade' observations are verifiable observations in which more than ^2^/3 of the observation’s identifiers agree on the organism’s identification (usually at species level). Research Grade observations are automatically shared with GBIF. The simplest method of an observation becoming Research Grade happens when the observer uploads a verifiable observation with a species-level identification (either user-supplied or chosen from iNaturalist’s machine learning suggestions), and another member of the iNaturalist community agrees with that identification. At that point, with two species-level identifications that are in agreement, the over two-thirds consensus threshold is crossed, and the observation is automatically marked as Research Grade. However, if a third community member then later adds a disagreeing identification, there is no longer an over two-thirds community agreement. Therefore, the observation is automatically moved back into the 'Needs ID' category.

It is important to note that, while making a verifiable observation is completely within the observer’s control (i.e., taking a photo or sound recording of a wild organism and ensuring the date and location are accurate), the criteria needed to move that observation to Research Grade only partially lies with the observer; for instance, making sure the photo or sound recording is clear so someone else can assess that evidence and provide an ID, or uploading it with an identification. Part of getting an observation to Research Grade is outside of the observer’s control, specifically the need for other members of the iNaturalist community to look at that observation and add identifications.

### 2.2 Participants and Settings

The current study explored the participation of 249 young people, aged 5−19 years old, on iNaturalist (this is the age range of young participants we previously found to participate in these types of programs). The research design consisted of two cohorts. The first cohort of Bioblitz participants (*n* = 135) joined at least one Natural History Museum Bioblitz (local field-based event), that used iNaturalist as the data-recording platform. The Bioblitzes were organised by the Natural History Museum of Los Angeles County or the California Academy of Sciences. Museums invited young volunteers to the Bioblitzes via social media (Facebook, Instagram and Twitter), on their websites, in newsletters, emails to previous attendees, and physical fliers/posters in the museum and museum events. The Bioblitz attendees were then invited to take part in this study, read an information sheet and complete a consent form. Participants in the second cohort (*n* = 76) were recruited via the internet (through a journal post on the 2019 City Nature Challenge umbrella project on iNaturalist, as well as via Twitter and the iNaturalist Discord server). Both cohorts may have had various levels of experience with iNaturalist prior to the study, including in-person training at Bioblitz or City Nature Challenge events. Participants were invited to provide us with more background information, such as their age group and gender. Background information and iNaturalist usernames were received from 135 participants. Participants' observations were publicly accessible and were retrieved from iNaturalist for the period between June 2013 and October 2020. In total, there were 196,832 observations recorded by this set of young people (both cohorts). The log files included information about each observation, such as usernames, date/time and location of the observation, taxonomic identification, whether the organism was captive/cultivated, and the quality grade of the observation. Prior to data analysis, the participant usernames were anonymised (e.g., iNat1, iNat2).

### 2.3 Tracking Young People’s Research Grade Observations

We used the proportions of the different types of data quality grades within iNaturalist to examine the extent to which young people contributed data that are potentially useful for biodiversity research. 'Needs ID' and 'Casual' observations were filtered out to allow us to focus on the 'Research Grade' contributions. Verifiable observations were calculated by adding the number of Research Grade and Needs ID contributions.

Stacked bar charts were used to visualise the comparisons of data quality grades between and within each iconic taxon category. The visualisations displayed the taxon categories with the most and least Research Grade classifications.

Research Grade ratio, the percentage difference between the Research Grade and the overall observations, was calculated for each participant. The closer to 1, the more Research Grade contributions are associated with a participant. A frequency graph was created to visualise the Research Grade ratio, supplemented by descriptive statistics. Data from all 249 participants were used to achieve RO1 (to explore the verifiability and quality grade of young volunteers' contributed organisms, overall and per iNaturalist’s taxon categories).

### 2.4 Participation Metrics

The participation metrics [6] that we adopted in this study are calculated as follows:

- Activity ratio is the ratio of days on which a user was active and contributed at least one observation in relation to the total days they remained linked to iNaturalist (from account creation to the last contributing day). The closer to 1, the more active a user is during the days they are linked to the platform. This metric provides us with information on how active a young volunteer is on iNaturalist.
- Relative activity duration is the ratio of days during which a user is linked to iNaturalist and to the total number of days from their first iNaturalist contribution day to the date that iNaturalist data were aggregated for this study (18 October 2018). The it is closer to 1, the longer a user remains on the platform. This metric provides us with information on the duration that a young volunteer is an active contributor on iNaturalist.

Research Grade ratio was then correlated to the participation metrics to indicate whether there is a relationship between the activity or duration to the levels of Research Grade contributions. Spearman’s Rho was used for the correlations because our dataset was not normally distributed. Data from all 249 participants were used to address RO2 (the relationship between participation behaviour and proportion of contributed Research Grade quality observations).

### 2.5 Background Characteristics

To explore how gender and age group relate to Research Grade ratio, independent samples t-test and analysis of variances (ANOVA) were performed. Scheffe post hoc tests confirmed flagged differences between groups detected by ANOVA. Data from participants who provided us with background information were used to explore differences between gender and age group. Descriptive statistics (Research grade ratio) used in the tests can be found in Table 1. An alpha level of 0.05 was used for all the analysis.

## 3 Results

In this section, we provide an overview of the participation of young people on iNaturalist, the results regarding their level and type of participation and the nature of their contributed observations.

### 3.1 Research Grade Observations (RO1)

Overall, the young volunteers contributed 111,907 observations that achieved the Reseorch Grade (58% of the total contributions by young people), 78,755 observations (40%) that remained classified as Needs ID, and 4125 (2%) that remained 'Casual' ebservations. The verifiable observations (Research Grade and Needs ID) of young participants encompassed 98% of their overall contributions.

**Figure 1:**
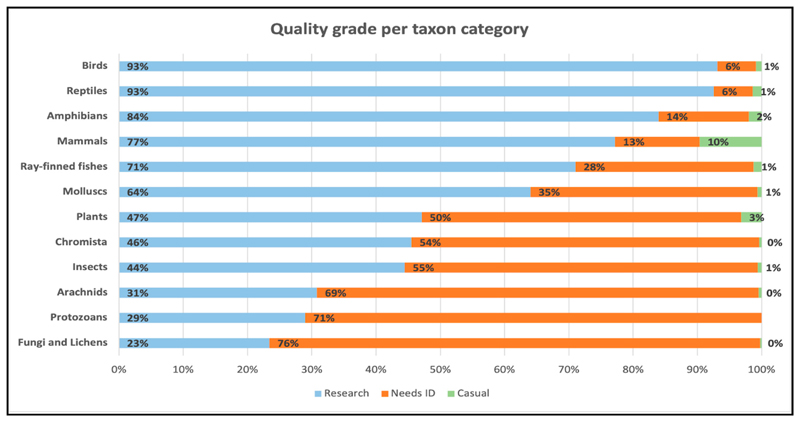
Quality grade per iNaturalist taxon category. The blue colour shows the percentage of youth’s contributions that became Research Grade; the orange colour shows the contributions that still need an ID; and the red colour represents the contributions that are Casual.

The taxon categories with the largest proportion of contributions achieving Research Grade (93%) were birds and reptiles (Figure 1). Other taxon categories with at least half of their contributions being labelled as Research Grade were amphibians (84%), mammals (77%), ray-finned fishes (71%) and molluscs (64%). The taxon category with the smallest proportion of Research Grade contributions was Fungi and Lichens (23%).

**Figure 2:**
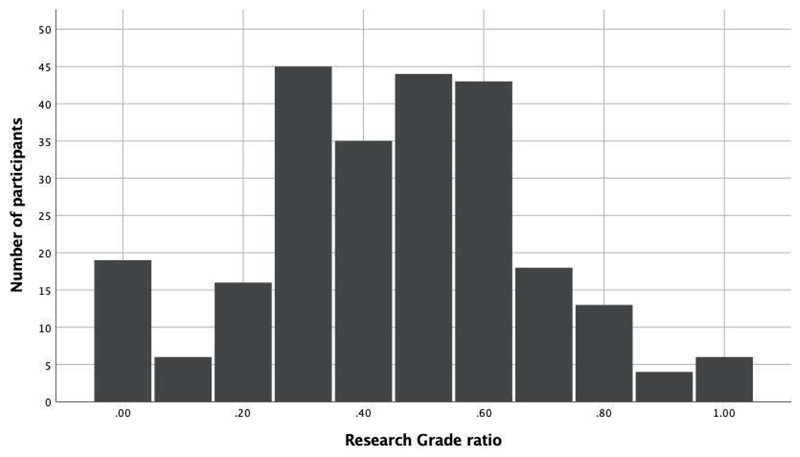
Frequency distribution of the Research Grade ratio. The graph shows the percentage difference between the Research Grade and overall observations. Youth contributed a median of 11 observations that became Research Grade.

Young participants contributed a median of 11 observations classified as Research Grade (ranging from 1 to 2599). Figure 2 shows the ratio of young people’s contributions classified as Research Grade to the total number of contributions (Research Grade ratio). The largest peak of young volunteers' Research Grade ratio is at 0.3 (*n* = 45), which means that three out of ten observations are of Research Grade quality. Furthermore, about two-thirds of the participants (67%) fall within the 0.3–0.6 Research Grade ratio. Among the study population, 7% had a very low Research Grade ratio (<0.1), and only a small minority scored a Research Grade ratio of more than 0.8 (4%).

### 3.2 Participation and Research Grade (RO2)

The results of the participation metrics (activity and duration) of participants were as follows:

- Activity ratio: *M* = 0.41, *SD* = 0.43;
- Relative activity duration: *M* = 0.43, *SD* = 0.43.

Young people, on average, were found to beactively contributing during 41% (*M* = 0.41, skewed right) of the days they were linked to iNaturalist (activity ratio). Moreover, they remained active contributors on iNaturalist for 43% (*M* = 0.43, U-shaped) of the total time that could be active (relative activity duration).

Results of the Spearman correlations Indicated that there were no statistically significant association between the Research Grade ratio of young volunteers and their activity (*r_s_* (249) = −0.09, *p* = 0.15). However, there was a positive significant association beiween the Research Grade ratio of young volunteers and their duration on the platform (*r_s_* (249) = 0.26, *p* < 0.01). Therefore, we reject the H1 null hypothesis (iNaturalist young volunteers' participation behaviour will not be related to the proportion of contributed Research Grade observations).

### 3.3 Youth Characteristics and Research Grade (RO3)

**Figure 3:**
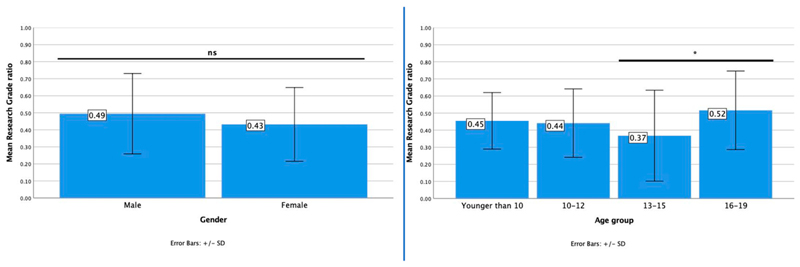
Research Grade ratio bar chart of means for gender (left) and age (right). The graph shows the Research Grade ratio mean and SD for each group and denotes significant differences, if any, between groups. The star (*) indicates significant statistical differences between groups.

Findings from examining the relationships among Research Grade ratio and background characteristics of young iNaturalist participants (Figure 3) indicate that there were no statistically significant differences between young people of different gender (*t* (119) = 1.52, *p* = 0.13). Therefore, we fail to reject the H2 null hypothesis (there will be no gender differences in the proportion of Research Grade observations of iNaturalist young volunteers).

However, the contributions of young volunteers aged 16−19 achieved a significantly higher Research Grade ratio than those aged 13−15 (*F* (3,131) = 3.17, *p* = 0.03); there was no statistically significant difference between the other age groups (Table 2). Therefore, we reject the H3 null hypothesis (there will be no age differences in the proportion of Research Grade observations of iNaturalist young volunteers).

The difference between young people aged 13−15 and 16−19 could be explained by various factors such as the quality of images and number of images per observation (multiple photos of different parts of an organism can improve the ability of others to identify it) they upload to the platform.

## 4 Discussion

### 4.1 Young Volunteers and Scientific Research in Biodiversity

Investigating young volunteers' observations that are 'verifiable' and those that have become Research Grade quality on iNaturalist provides us with insights into the extent to which they can potentially contribute to scientific research, and which fields of science can benefit from their contributions (RO1).

We found that each young volunteer contributed on average 11 observations that became Research Grade, with a large proportion contributing only a few Research Grade observations and only a few contributing a large number of Research Grade observations. These results extend our current knowledge of asymmetrical participation patterns (e.g., Ponciano and Brasileiro, 2015) to asymmetrical Research Grade quality contribution patterns.

Compared to the general iNsturalist contributed observations that achieved Research Grade across all participants (54%), young people contributed more observations that became Research Grade (58%), but not by much. This means that, in fact, young people’s observations in this study were proportionally similar in data quality as the broader iNaturalist community consisting primarily of adults. However, it is interesting to note that verifiable contributed observations made by young people in this study (those that include a valid date, location, photo/sound, and are not captive/cultivated) make up a much larger proportion (98%) of their overall contributions: 10% more than the overall iNaturalist verifiable contributions (88%) [38]. We cannot assess whether this difference is statistically significant or rather random. However, we can say for the context of this study that the young people were most often facilitated in some way in their introduction to iNaturalist, through a BioBlitz event or a program in which they would have received training on how to take usable photographs and make verifiable observations. Just over 69% of youth in this study came from Bioblitz events run by our museums. Therefore, future studies should seek to clearly establish whether young volunteers are more likely to contribute verifiable observations than the greater iNaturalist community, and by extension, which types of projects and programs best support youth in this endeavour. This is important because while becoming Research Grade is required to be shared with GBIF, any verifiable contribution has the potential to become Research Grade.

With regard to evaluating the potential usefulness of young participants' contributions, we suggest that researchers across all taxa, but especially those investigating less frequently, and having identified more diverse taxa (such as fungi and lichens, protozoans, molluscs, insects, and arachnids) may benefit from young volunteers' contributions (and in fact, all contributions to iNaturalist). This can be done by actively curating and identifying those observations that achieve Research Grade through concerted CCS project efforts or just by spending time identifying observations on the site. Since 2015, there have been 1203 publications resulting from data first shared on iNaturalist, accessed through GBIF (GBIF iNaturalist Dataset page). Biodiversity researchers with taxonomic expertise can increase the number of Research Grade observations that are shared with GBIF, ensure Research Grade observations that are shared are correct, and become an active part of a thriving naturalist community, by identifying people’s observations and moving them to Research Grade. For example, researchers at the Natural History Museum of Los Angeles County have active reptile/amphibian, and snail/slug projects in which scientists identify local observations to increase the likelihood that observations become Research Grade. Similarly, researchers at the California Academy of Sciences continually identify observations made along the California Coast in order to ensure that as many observations as possible are Research Grade. These data are then used to build tools to improve management and understand the effects of climate on marine species [39].

It is, however, interesting to note that the most observed organisms by young people in a previous study on iNaturalist [12] are plants and insects, while birds and reptiles (which constitute the majority of youth’s Research Grade observations in this study) are only a small proportion of their overall contributions. The reasons for this result are not yet entirely understood but can potentially be explained by the difficulty in photographing particular organisms (e.g., flying birds and fast-moving reptiles) without specialised camera equipment [27]. It is relatively easy to find and photograph plants as they are non-moving, and due to their abundance, it is generally easy to find some groups of insects (e.g., ants, some beetles, bees). However, youth do not always take photos of the key characteristics needed, i.e., length of plant stems (lack of training), or cannot get an in-focus photo (lack of necessary camera zoom/magnification equipment). Furthermore, whether a young person’s (or any person’s) observation achieves Research Grade is also closely connected to the number of people in the larger iNaturalist community who have an interest in identifying or the ability to identify particular taxa have familiarity with species in particular geographic regions. Overall, birds, mammals, reptiles and amphibians are more rapidly identified on iNaturalist relative to other taxa: for example, looking at all the observations that were uploaded in 2020, as of the time of writing, the taxa with the greatest proportion of observations that are now Research Grade are birds (94%), reptiles (91%), mammals (85%), and amphibians (81%), while taxa with a greater number of species and/or less easily identifiable species are much lower, like plants (56%), insects (53%), and fungi (27%) [40]. Regions with more active users also have their observations identified sooner than observations from less active regions [41].

Overall, our findings are consistent with previous results on young people’s ability to capture biodiversity images that can be identified and reach Research Grade by the iNaturalist community, and which are of potential scientific use [28]. We also suggest our findings resonate with earlier findings [29] on the importance of skills training in order to involve young volunteers in CCS activities. We suggest that various forms of training (e.g., photography techniques, including accurate date/time/geographic location metadata, species identification skills) need to be emphasised during programmes where volunteers of any age are participating in biodiversity CCS projects utilising app-based data collection platforms.

### 4.2 Youth Participation Behaviour and Characteristics

A significant outcome from studying the participation behaviour of the iNaturalist young volunteers was that those with longer participation, in particular a longer period of being registered with iNaturalist, have a greater proportion of their observations that have become Research Grade (RO2). Thus, they are more likely to contribute to biodiversity research. This positive association between length of participation and Research Grade ratio corroborates previous research connecting adult participation with increased numbers of correct organism identifications [26] and prior knowledge or repetition of method [23], and positive correlation between the volunteer accuracy of data collection correlated with the persistence of participation in the project [19]. However, part of the process of an observation becoming Research Grade is beyond the observer’s control and relies on other community members identifying observations. Therefore, it is likely that one of the drivers of this finding is that the longer each observation is on iNaturalist, the greater the chance that it has been seen by someone who knows how to identify it, has been identified and has become Research Grade. For example, 59.1% of all verifiable observations uploaded in 2020 are now Research Grade, 62.8% of all verifiable observations uploaded in 2019, 66.2% of all verifiable observations uploaded in 2018 and 70.1% of all verifiable observations uploaded in 2017 [38]. The importance of an observation’s duration on the platform is especially true for those that were not uploaded with a species-level identification, which need more than one community member to see it, know how to identify it and provide an identification, or an observation where a community member has disagreed with what the identification is and thus needs more members identifying it to reach the more than ^2^/3 consensus threshold to reach Research Grade. However, in Scanlon et al. [26] and Lewandowski and Specht [23], participation is measured in number of contributions. In the current study, our significant result relates to longer participation or longer time on the platform, regardless of whether the young person continues to be actively participating. Further research is needed to better understand the interplay between the contributions of young people, their learning and skill development, in addition to the role of the community in this process. This finding also leads to important questions for future research about the relationship between participation, learning, and potentially useful contributions to science.

In response to RO3 and the relationship between young volunteers' characteristics and Research Grade observations, we found that, in contrast with earlier findings on adults, there were no significant differences between male and female participants [23]. Our results may be explained by findings on young people in STEM [42], indicating that girls are equally or more likely than boys to achieve minimum proficiency levels in STEM; however, fewer girls than boys aspire to careers in STEM, even among top performers. There was, however, a significant difference between young people aged 13-15 and 16-19, with the latter contributing observations that become Research Grade to a greater extent. The reason for this result is still not entirely clear, but it could be attributed to unknown external factors. For example, it could be explained by older youth having access to specialty equipment to better observe birds (e.g., a DSLR camera with telephoto lenses), or a general interest of children in particular age groups for certain species. It could also be explained by youth’s prior training [23] in terms of observing organisms as part of a natural history museum program, a school class activity, or with the guidance of their parents or guardians.

### 4.3 Limitations

The present study has only examined the observations of young volunteers on iNaturalist generally linked to three Natural History Museums (two in California and one in the UK). Hence, any outcomes should be discussed within the context of this study and interpreted with caution, rather than being generalised to the population. It is also noted that this is an exploratory study aiming to identify the potential relationships between Research Grade, participation and background characteristics. In the future, this work should be replicated and include additional factors that have shown to relate to verifiable and Research Grade observations, including photograph quality and the crowdsourcing effort for data verification [27]. Future work should focus on the factors that could increase and explain the activity, duration, and systematic contribution as well as the quality of contributions of young volunteers. This future work could involve investigating how young people learn by observing or identifying organisms on iNaturalist and the associations between young volunteers and self-identified comfort levels [25] with participation and contributions.

## 5 Conclusions

This study was the first step towards enhancing our understanding of how young volunteers contribute to biodiversity science through iNaturalist. We found that young people contribute observations that achieve Research Grade quality. Our work also broadens the knowledge of asymmetrical participation in CCS by providing evidence of asymmetrical research quality grade contribution. The majority of young participants contribute a small number of observations that become Research Grade and just a few participants contribute the majority of observations that become Research Grade.

Perhaps most importantly, the evidence from this study suggests that young people with lasting participation on iNaturalist are more likely to contribute observations that become Research Grade (and therefore more accessible and potentially useful to biodiversity research and monitoring). Our findings add to a growing body of literature on participation and data quality in online CCS. Future work, including more qualitative approaches to studying young volunteers' learning and engagement, could inform our understanding of what stimulates and supports contributions that are potentially useful to science. It could also help us understand how these factors can be reinforced by platform design and/or museum and other CCS practitioners.

Given our discussion above of whether something becomes Research Grade depends both on the behaviours of the young contributor and the actions of the iNaturalist community, our findings have implications for both of these audiences in enhancing biodiversity science. Our findings do raise interesting questions as to how young volunteers' observations achieving Research Grade relate to their skills of photographing a particular organism and/or the community’s interest in identifying this organism. Taken together, our findings have implications for the design of CCS citizen science programs broadly and those using iNaturalist specifically.

While we did not explicitly examine in this paper the extent to which facilitation, training and external supports may have influenced data quality, our findings do reveal some clear areas where good conservation and education outreach techniques [43], and increased attention from biodiversity researchers, could increase the likelihood that volunteers' iNaturalist observations are verifiable, regardless of their age. To increase biodiversity observations available for research and better support volunteers, young or old, in iNaturalist-based CCS projects, we recommend:

- Biodiversity scientists and others with taxonomic expertise interested in using iNaturalist observations in research need to identify verifiable observations to help move them to Research Grade, and to update and improve the identification of observations that are currently Research Grade;
- CCS project managers need to design projects that work with their target audience’s skill level in mind, build in data quality assurance and control procedures, and continue engagement to help encourage lasting participation on iNaturalist;
- CCS project managers need to provide adequate training and equipment (e.g., magnification clips for phones) to move them towards making verifiable observations—which includes tips for taking identifiable photographs for science and how to check date, time and geographic location.
- If involving young people is a goal, CCS project managers and biodiversity scientists need to work together (although we recognise that sometimes they are one in the same group of people) to ensure youth are fully supported in their efforts to take part in iNaturalist-based CCS projects.

Overall, this study has shown that youth do submit observations to iNaturalist that have the potential to inform research. Providing scaffolding to the youth, such as in-person engagement, training on using iNaturalist, and tools for taking better photographs, as well as biodiversity scientists and amateur experts working to move verifiable observations to Research Grade will likely enhance the extent to which young people contribute to biodiversity science.

## Figures and Tables

**Table 1:** Background characteristics and Research Grade (RG) ratio descriptive statistics

Background Characteristics	N	RG Ratio Mean (M)	RG Standard Deviation (SD)
Gender			
Male	55	0.49	0.24
Female	66	0.43	0.22
Age group			
<10	22	0.45	0.17
10−12	17	0.44	0.20
13−15	34	0.37	0.27
16−19	62	0.52	0.23

**Table 2:** ANOVA post hoc comparisons of Research Grade ratio and age groups.

	Research Grade Ratio			Scheffe Comparisons (*p* Value)		
Age Groups	*n*	M	SD	<10	10−12	13−15
1. <10	22	0.45	0.17	-	-	-
2. 10−12	17	0.44	0.20	1.00	-	-
3. 13−15	34	0.37	0.27	0.58	0.76	-
4. 16−19	62	0.52	0.23	0.76	0. 70	0.03*

Note: * p < 0.05.

## Data Availability

The data presented in this study are openly available on Figshare at https://doi.org/10.21954/ou.rd.14932131 (accessed on 1 February 2021).
